# Interpretation of p53 immunohistochemical stain of TP53-mutated myeloid neoplasms

**DOI:** 10.3389/fonc.2026.1916425

**Published:** 2026-08-14

**Authors:** Nawroz Barwari, William Patrick Morrow, Joni K. Evans, Nancy Rosenthal, Michael W. Beaty, Eric Hsi, Giovanni Insuasti-Beltran, Danielle L.V. Maracaja

**Affiliations:** 1 Department of Pathology, Wake Forest University School of Medicine/Atrium Health Wake Forest Baptist Medical Center, Winston-Salem, NC, United States; 2 Department of Biostatistics and Data Science, Wake Forest University School of Medicine, Winston-Salem, NC, United States; 3 Department of Laboratory Medicine and Pathology, Mayo Clinic, Rochester, MN, United States; 4 Department of Pathology, Duke University, Durham, NC, United States

**Keywords:** diagnostic accuracy, interobserver agreement assessment, p53 immunohistochemistry (p53 IHC), p53 protein expression, *TP53* mutation and expression, *TP53*-mutated myeloid neoplasms

## Abstract

**Background:**

*TP53* mutational status (*TP53*ms) is prognostically important in myeloid neoplasms. Immunohistochemistry (IHC) of p53 has been used as a surrogate for *TP53* mutations, with faster results than next-generation sequencing (NGS) and cytogenetic analysis. Given the challenges posed by heterogeneous p53 IHC expression, this study aims to evaluate the interobserver interpretation of p53 stain as a surrogate marker for *TP53m*.

**Methods:**

Fifty-three bone marrow (BM) specimens previously diagnosed as myeloid neoplasms or nonneoplastic BM specimens with available concurrent *TP53ms* were evaluated. p53 IHC staining was performed on all 53 core biopsy or clot sections. *TP53* mutation was found in 24 cases (*TP53*m). Twenty-nine specimens were *TP53* wild-type and served as the control group. Six hematopathologists (HPs) blinded to the diagnosis and *TP53*m independently scored the p53 staining pattern of the biopsies/clot sections for overall staining percentage (OSP) and staining intensity (dark staining percentage as a percentage of total cells (DCP)).

**Results:**

The intraclass correlation was excellent for OSP (0.927) and good for DCP (0.848). For OSP, a cutoff of 30% resulted in sensitivity of 83.3%, specificity of 89.7%, PPV of 87.0%, and NPV of 86.7%. For DCP, a cutoff of 10% achieved sensitivity of 87.5%, specificity of 96.6%, PPV of 95.4%, and NPV of 90.3%. Additionally, DCP significantly correlated with the VAF of *TP53*m (R = 0.469, p = 0.028).

**Conclusion:**

High inter-rater reliability for OSP and DCP supports integrating p53 IHC into bone marrow evaluations for rapid *TP53*m assessment. With superior specificity and predictive values, DCP proves to be a more reliable diagnostic marker for *TP53* mutations. While this approach shows promise for myeloid neoplasms, further validation is needed to overcome challenges and expand its clinical utility for tailored treatment strategies.

## Introduction

*TP53* is a gene located on chromosome 17 that is involved in cancer surveillance and encodes the tumor suppressor protein P53. It maintains the integrity of the cellular mechanisms associated with DNA repair, cell cycle regulation, and apoptosis (1–5). Detection of mutations in the *TP53* gene is critical for the diagnosis and prognosis of many hematopoietic and nonhematopoietic neoplasms. These mutations are seen in 7-11% of all myelodysplastic neoplasms (MDS) (6) and approximately 7% of all acute myeloid leukemia (AML) (7), with a higher percentage of those that are therapy-related or have complex cytogenetics (53–78%). The biallelic or multihit *TP53* gene loss-of-function is a diagnostic criterion of acute erythroid leukemia and myeloid neoplasms with associated *TP53* inactivation (6). *TP53* mutations are independently associated with resistance to chemotherapy, lower complete remission rates, and shorter overall survival (1–4, 8).

As molecular and cytogenetic tests are rarely immediately available during morphologic evaluation, p53 immunohistochemistry (IHC) has been explored as a tool to predict *TP53* mutation status (*TP53*ms) (4, 8). However, the interpretation of p53 IHC is complex and heterogeneous, as non-neoplastic marrow can exhibit weak P53 protein expression in hematopoietic elements with wild-type *TP53* (3). Although some studies have compared IHC with molecular testing, inter-observer variability has not been thoroughly assessed.

The study investigated p53 immunostaining patterns in bone marrow (BM) samples from cases with *TP53* mutations and wild-type *TP53* (a control group for comparison), as previously characterized by next-generation sequencing (NGS) and cytogenetics. Inter-observer variability in staining interpretation among six hematopathologists was also assessed, focusing on different staining patterns and the percentages of both overall and dark-stained cells. While prior studies have correlated BM p53 IHC results with *TP53ms* and cutoff values for interpretation (2, 4, 8, 9), they did not address inter-observer variability in a blinded manner. Additionally, some studies have relied on digital estimation methods, which are not widely accessible in all diagnostic laboratories.

## Materials and methods

Fifty-three BM specimens were collected over 14 months with concurrent NGS analysis from the Atrium Health Wake Forest Baptist Hospital (AHWFB). Twenty-four cases had *a TP53* mutation (*TP53*m) (monoallelic, biallelic, or multi-hit), and 29 were negative for *TP53* mutation (Wild type *TP53;* WT-*TP53*). The latter served as the control group.

The p53 stain IHC was performed 40 decalcified bone marrow core biopsy specimens and 13 non-decalcified clot section specimens. Six hematopathologists (HPs) independently reviewed the stains and estimated the overall percentage of positive cells (overall staining percentage, OSP) and the percentage of cells with strong nuclear staining (dark cells percentage, DCP). Cells were scored as <5%, 5%, 5-10%, and then in increments of 10%. The HPs were blinded to diagnosis, *TP53ms* and clinical details. The control group included non-neoplastic bone marrow cases and cases with myeloid neoplasms with WT-*TP53*. A plasma cell neoplasm was also included to match a plasma cell neoplasm in the *TP53*m group. The percentages were compared with those of *TP53*m and the diagnosis.

The results were shared between participants and evaluated by a biostatistician. This study was reviewed and approved by the AHWFB institutional review board.

### Immunohistochemical staining

Immunohistochemical staining was performed on formalin-fixed, paraffin-embedded BM trephine core biopsies and clot sections sectioned at 4-μm. Trephine core biopsies were submitted to Calfor Fixative Decalcifier (Cancer Diagnostics, Inc., Durham, NC, USA). A p53 monoclonal antibody DO-7 (PAOO57 ready-to-use, Leica Biosystems, Newcastle, UK) was used on Leica Bond 3 immunostainer (DS9800 Bond Polymer Refine DAB detection system, Leica Biosystems, Newcastle, UK) according to the manufacturer’s instructions. This monoclonal antibody recognizes the wild-type and mutant forms of the human P53 protein.

### Genomic profiling by next-generation sequencing

Libraries were prepared from 10 ng of DNA and RNA per pool and sequenced using the Oncomine Myeloid Assay GX v2 panel (Myeloid GX v2, catalog number A50694, Thermo Fisher Scientific) on the Ion Torrent Genexus Integrated Sequencer (catalog number A45727, Thermo Fisher Scientific) using the Ion Torrent GX5™ semiconductor chip (catalog number A40269), according to the manufacturer’s instructions. Myeloid GX v2 targets a comprehensive panel of DNA mutations and RNA fusion transcripts including 17 complete genes (including *TP53*), 28 hotspots, and 30 fusions covering all major myeloid neoplasms. Library preparation, template preparation, and sequencing were performed automatically by the Ion Torrent Genexus Integrated Sequencer. NGS data was analyzed using Genexus software version 6.6.2.1. Variant calling was performed using Myeloid GX v2 for GX5 (DNA and fusions) w4.2.2 workflow. A review of the identified variants was performed using the Oncomine Variants (5.16) and Oncomine Extended (5.16) integrated filters, and an unfiltered review of all samples. Sequencing quality control metrics were as follows: DNA mapped reads ≥ 950,000, mean read length ≥ 200 bp, coverage uniformity ≥ 97%; RNA fusion panel mapped reads ≥ 150,000, and mean read length ≥ 80 bp.

### Karyotype

A conventional chromosome analysis was performed. The bone marrow aspirate sample was cultured, and G-banded cytogenetic analysis was performed on metaphase spreads from methanol/acetic acid-fixed cultured cells. Analysis and karyotyping of captured images representing 15 colonies or 20 cells were performed by two technologists; additional metaphases were assessed when indicated. The results were documented in accordance with the International System for Human Cytogenetic Nomenclature.

### Biostatistics

Basic characteristics of the subjects were summarized as either mean ± standard deviation (age) or number and percentage and were compared using a t-test (age) or Fisher’s exact test. Interobserver reliability among the six HPs was measured using the Fleiss kappa (for intensity and a dichotomized measure of OSP and DCP) or the Shrout-Fleiss intraclass correlation coefficient (ICC) for the percentage of OSP and DCP, providing insight into the level of agreement among observers. Values closer to 1 indicated high agreement, while values closer to 0 indicated poor agreement. The ICC was supplemented with box plots to illustrate the spread of ratings; the boxes showed the interquartile range (IQR), 25th to 75th percentiles, as well as the 10th, 50th, and 90th percentiles. OSP and DCP were averaged across the pathologists, the median value across the pathologists was determined, and these summary statistics were compared between those with and without the gene mutation using the Wilcoxon rank-sum test.

The Pearson correlation coefficient for OSP and DCP with the Variant Allele Frequency (VAF) was estimated, and the hypothesis that the correlation was equal to zero was tested. These correlations were estimated for the percentage determined by each hematopathologist individually, as well as for the average and median across the six HPs. Logistic regression models were fitted to predict the presence of a *TP53* mutation separately using the median of the six HPs estimates of OSP and DCP. From each model, the area under the receiver operating characteristic (ROC) curve was estimated, and Youden’s index was calculated to determine the value of either OSP or DCP that maximized sensitivity and specificity. Based on these values, sensitivity, specificity, positive predictive value (PPV), and negative predictive value (NPV) were calculated. All analyses were performed using SAS software, version 9.4, SAS Institute, Cary, NC.

## Results

### Study case characteristics

This study evaluated a cohort of 53 specimens based on *TP53*ms. Twenty-nine specimens were WT-*TP53* (control group), and 24 cases had mutated *TP53* (monoallelic, biallelic, or multi-hit) identified through NGS. The median age was 68 (range 29-94), with a male-to-female ratio of 2.53. Patients with a *TP53* mutations were slightly older (p=0.052) and more often male (p=0.005). The karyotype was also evaluated. A complex karyotype was significantly more frequent in the *TP53* mutation group (83.3%) compared to the no mutation group (3.5%) (p < 0.0001). Conversely, a normal karyotype was predominant in the no mutation group (96.5%) compared to the *TP53* mutation group (16.7%).

Of the 24 *TP53*m cases, 11 cases were diagnosed as MDS. From the MDS cases, nine showed biallelic *TP53* mutations (two post-cytotoxic therapy). Most MDS cases (6/9) showed increased blasts (10-19%) and were further classified as MDS/AML according to the International Consensus Classification 2022 (ICC2022) or MDS with biallelic *TP53* inactivation By World Health Organization 5^th^ Edition. Nine cases were diagnosed as AML, seven of which were myelodysplasia-related according to the WHO 5^th^ edition. Of these, six were additionally classified as AML with mutated *TP53*, according to ICC2022, and one was AML with myelodysplasia-related cytogenetic abnormalities. Of the remaining AML cases, one case was AML post-cytotoxic therapy, with *KMT2A* rearrangement (WHO 5^th^ edition classification)/AML, therapy-related, with mutated *TP53*, and with *MLLT3::KMT2A* (ICC2022 classification), and the last case was AML with KMT2A rearrangement according to WHO 5^th^ Edition/ICC2022 classification. Other cases included in the *TP53* mutated group: one case of chronic myelomonocytic leukemia-1 (CMML-1), myelodysplastic subtype by both WHO and ICC2022 classifications; one case with myelodysplastic/myeloproliferative neoplasm with neutrophilia (also known as atypical chronic myeloid leukemia by ICC2022); one case in a patient with a history of acute monocytic leukemia in remission (initially *TP53* negative) showed a new low-level *TP53* mutation (VAF 2.35%); and one case of clonal hematopoiesis of indeterminate potential in a patient with plasma cell neoplasm.

The disease states varied significantly within the control group including myeloid and nonmyeloid neoplasms. Eighteen cases were negative for a myeloid process; eight of which had an unremarkable marrow with nonspecific symptoms in patients with a previous history of myeloid malignancy status post-stem cell transplant or chemotherapy. Three cases were diagnosed as AML; AML without maturation, AML with *NPM1* mutation, and AML MDS-related. One case was diagnosed as myeloid sarcoma with no involvement in the bone marrow. Other cases included: MDS with low blasts and multilineage dysplasia, MDS with low blasts and 5q deletion, plasma cell neoplasm (n=1), CMML (n=1), myeloproliferative neoplasm (MPN) (n=1), systemic mastocytosis (n=1), monoclonal gammopathy of undetermined significance (n=1), and T-cell clonal populations of undetermined significance.

Among the *TP53*m cases, three exhibited a normal karyotype (3/24), whereas the remaining cases displayed a complex karyotype (21/24). Ten of the 24 *TP53*m cases exhibited deletions in chromosome 17 on karyotyping. Conversely, of the 29 cases in the WT-*TP53*, only one displayed a complex karyotype (MDS with low blast and multilineage dysplasia, WHO 5^th^ Edition).

Missense mutations were predominant among all *TP53*m cases, except for one that featured a frameshift mutation (AML, myelodysplasia-related). Biallelic mutations were present in 15 cases, defined as biallelic mutations, variant allele frequency >50%, and/or karyotype with 17 deletions). Loss of heterozygosity were not evaluated in this study. Monoallelic mutations were present in nine cases. Seven cases, including diagnosis of AML (3), MDS (3) and MDS/MPN (1), showed hotspot mutation in p.(R175H), p.(R273H), p.(R273C), and p.(G245S).

### *TP53* IHC staining evaluation and inter-observer agreement

The study included 40 decalcified bone marrow core biopsy specimens and 13 clot section specimens. No apparent differences in staining quality or interpretation were observed between the two specimen types during review of the study cohort.

**Figure 1:**
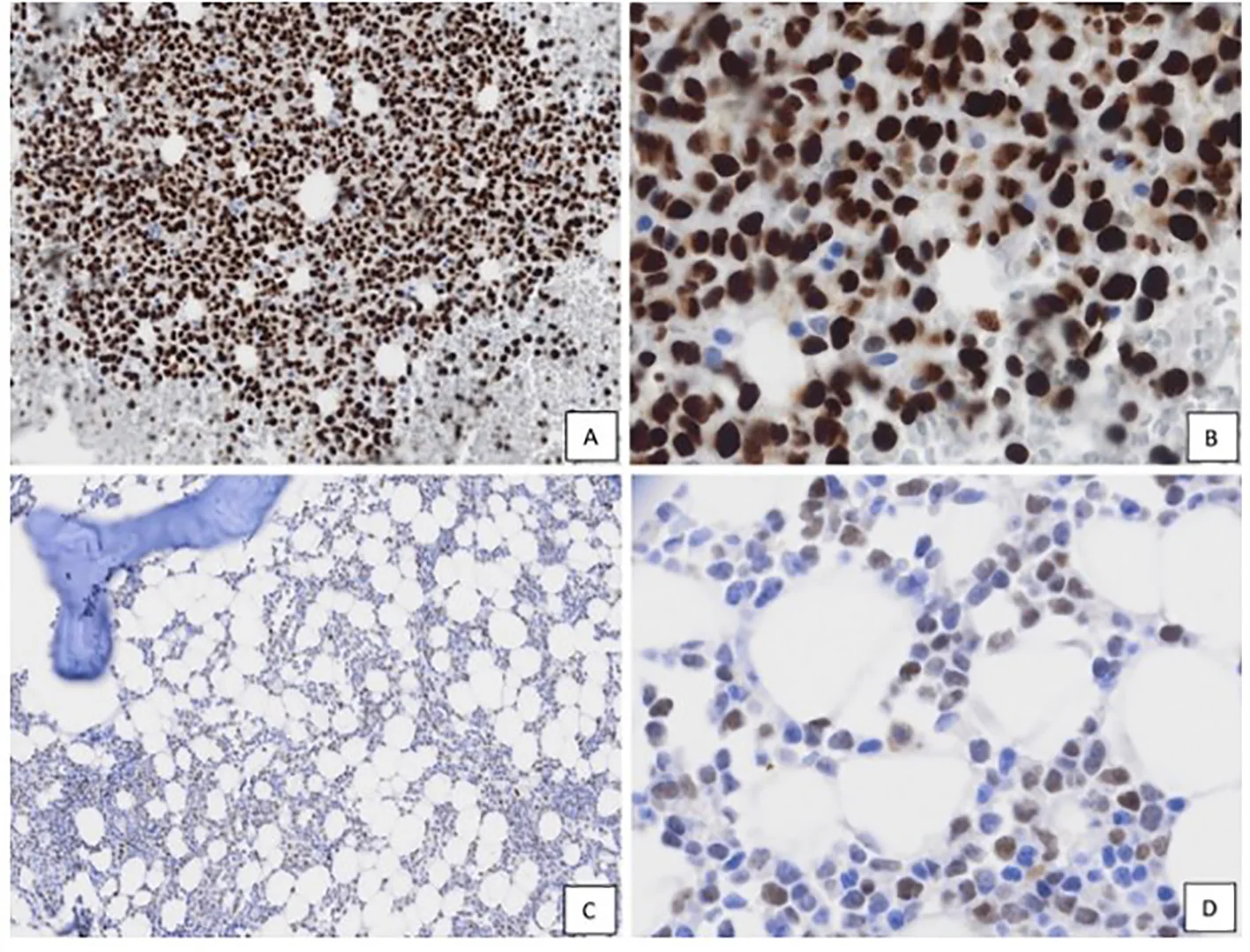
**(A, B)** Example of AML post-cytotoxic therapy with TP53 mutation (p.(M237I), c.711G>A, and VAF 94%) demonstrating strong p53 expression by immunohistochemistry (8x and 20x digital image, respectively). Panel **(C, D)** is an example of a case of AML, myelodysplasia-related, with TP53 wild type (4x and 20x digital image, respectively).

**Figure 2:**
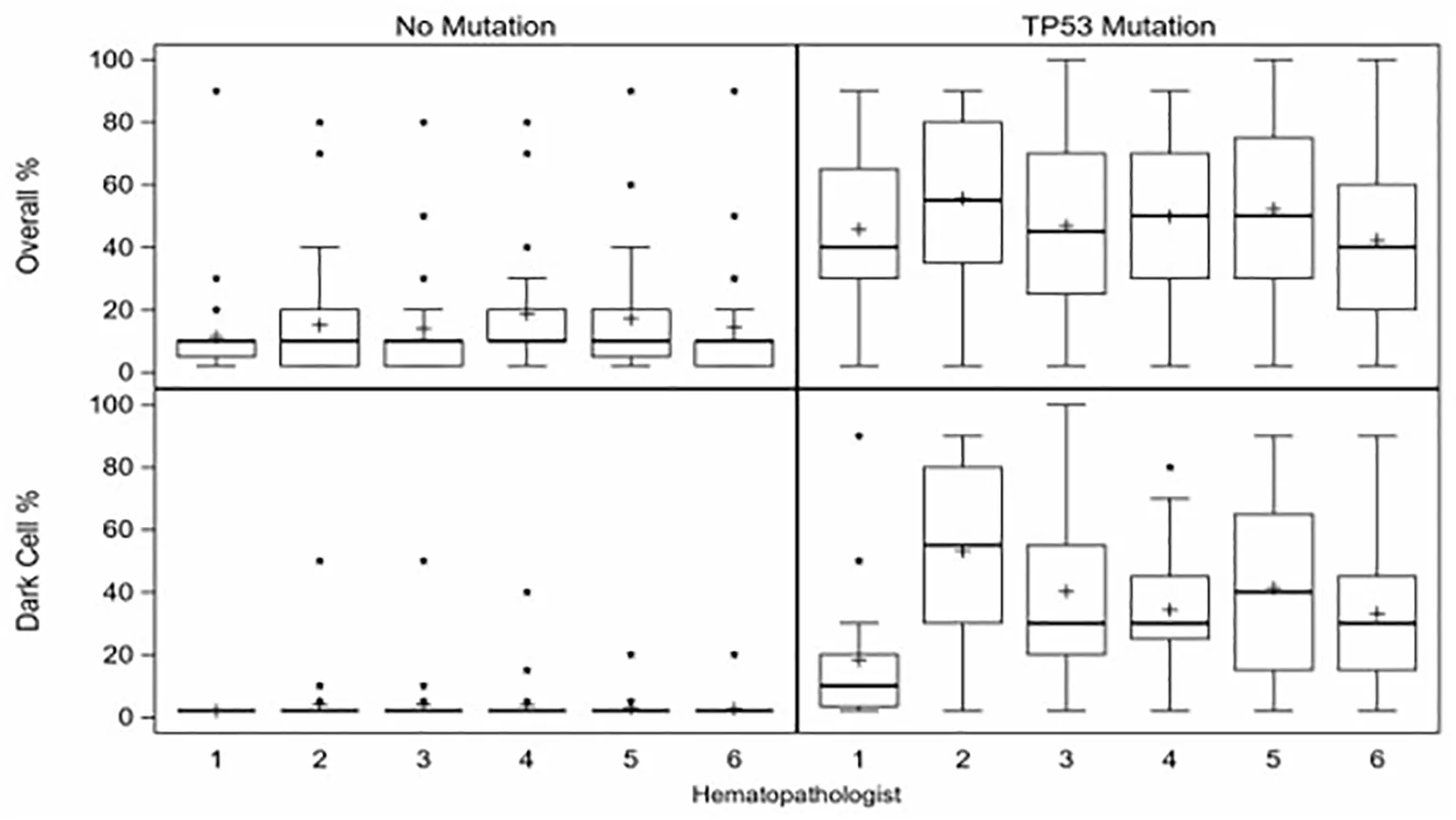
Box plots for overall staining percentages (OSP) and dark cell percentages (DCP) among the 6 hematopathologists.

Each pathologist independently evaluated the staining characteristics of samples from the *TP53*-mutated and WT-*TP53* groups according to predefined p53 IHC scoring criteria (**Figure 1**). **Figure 2** illustrates the distribution of overall staining positivity (OSP) and dark cell positivity (DCP) scores across pathologists, stratified by *TP53* mutation status, and demonstrates the overall consistency of scoring within each group.

Agreement among the six hematopathologists was assessed using three complementary measures: the intraclass correlation coefficient (ICC) for continuous scoring, Fleiss kappa for dichotomized categorical scoring, and exact agreement to evaluate complete concordance among observers.

#### ICC (continuous agreement)

The ICC was used to assess agreement for the continuous estimates of OSP and DCP. Across all cases, regardless of *TP53* mutation status, agreement was excellent for OSP (ICC = 0.927) and strong for DCP (ICC = 0.848). When stratified by *TP53* mutation status, ICC remained high for OSP (0.878 in WT-TP53 cases and 0.905 in TP53-mutated cases) and substantial for DCP (0.644 and 0.806, respectively), indicating robust interobserver agreement for both measures (**Table 1**).

**Table 1:** Measures of agreement for both overall staining percentage and dark cell percentage.

Cohorts	Shrout-Fleiss ICC		Exact agreement (<5% vs >5%)		Exact agreement (<10% vs >10%)	
	*OSP*	*DCP*	*OSP*	*DCP*	*OSP*	*DCP*
Overall	0.927	0.848	35/53 (66.0%)	45/53 (84.9%)	35/53 (66.0%)	46/53 (86.8%)
With *TP53* Mutation	0.905	0.806	23/24 (95.8%)	21/24 (87.5%)	23/24 (95.8%)	20/24 (83.3%)
No Gene Mutation	0.878	0.644	12/29 (41.4%)	24/29 (82.8%)	12/29 (41.4%)	26/29 (89.7%)

#### Fleiss kappa (categorical agreement)

Fleiss kappa was used to evaluate categorical agreement after dichotomizing OSP and DCP using predefined cutoff values (<5% vs ≥5% and <10% vs ≥10%). Both cutoff strategies demonstrated moderate-to-substantial agreement among observers, with consistently higher agreement for DCP than for OSP (**Table 1**).

#### Exact agreement

To complement these analyses, exact agreement among the six hematopathologists was calculated for the dichotomized categories. Exact agreement was highest for DCP, particularly among *TP53*-mutated cases, further supporting the reproducibility of DCP assessment (**Table 1**).

### *TP53* IHC stains predict *TP53*ms

**Figure 3:**
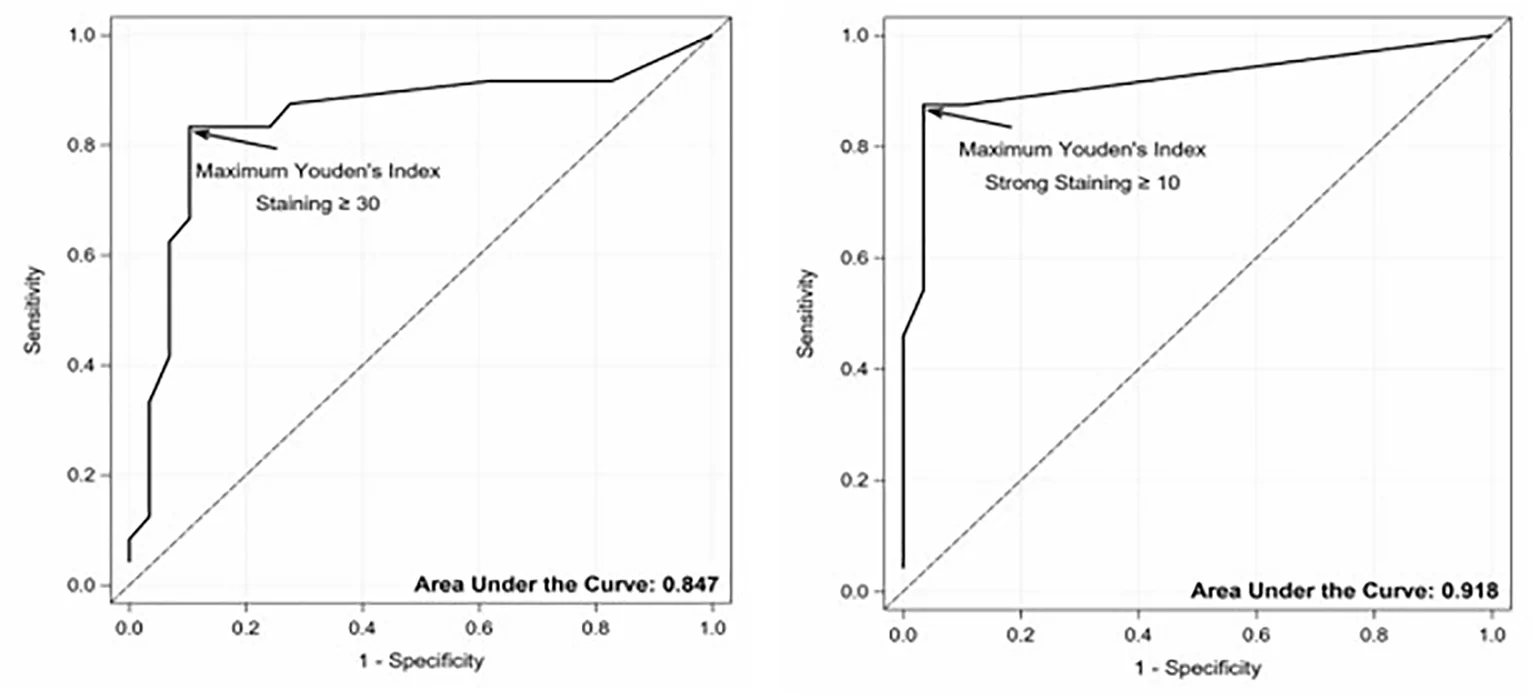
P53 IHC as a predictor of TP3 mutations inactivation: receiver operating characteristic (ROC) curves for overall staining percentage (left) and dark cells percentage (right). The median score among the 6HP for both OSP and DCP was used for ROC analysis.

The diagnostic performance, including the sensitivity, specificity, and predictive values for OSP and strong nuclear DCP staining in predicting *TP53* gene mutations were evaluated. Two ROC curves were analyzed, one for OSP and one for DCP. Both ROC curves demonstrated a very good area under the curve (AUC), indicating a high diagnostic accuracy (**Figure 3**). When applying a 30% cutoff for OSP, the sensitivity was 83.3% and the specificity was 89.7%, with a PPV of 87.0% and a NPV of 86.7%. For the DCP, when applying a 10% cutoff, the sensitivity was 87.5%, and the specificity was 96.6%, with a PPV of 95.4% and the NPV of 90.3%. These results highlight that both OSP and DCP effectively predict *TP53* gene mutations, with DCP≥10% demonstrating slightly higher sensitivity, specificity, and predictive value than OSP≥30%.

Similarly, the OSP and DCP were compared across *TP53*m both overall and within each diagnostic group (**Table 2**). Both measures showed significant differences (p<0.0001) between cases with and without the TP53 mutations. Specifically, for patients diagnosed with MDS, both OSP and DCP were significantly higher in those with the mutation. In contrast, among patients diagnosed with AML or other myeloid neoplasms (non-MDS), staining did not differ significantly by mutation status. These findings provide important insights into the relationship between staining characteristics and mutation status across different diagnostic categories.

**Table 2:** Characteristics based on mutation status.

Characteristics	Total (n= 53)	No mutation (n=29)	TP53 mutation (n=24)	P-value
Age (years), mean ± SD	65.8 ± 14.5	62.3 ± 14.1	70.0 ± 14.0	0.0521
Age (years), median (range)	68 (29 – 94)	64 (39 – 94)	75.5 (29 – 86)	
Gender, No. (%)				0.0051
Female	15 (28.3%)	13 (44.8%)	2 (8.3%)	
Male	38 (71.7%)	16 (55.2%)	22 (91.7%)	
Karyotype, No. (%)				<0.0001
Complex	21 (39.6%)	1 (3.5%)	21 (87.5%)	
Deletion of chromosome 17	10 (18.9%)		10 (41.7%)	
Normal	32 (60.4%)	28 (96.5%)	3 (12.5%)	
TP53 Mutation type, No. (%)				
Missense	23 (43.4%)		23 (95.8%)	
Monoallelic	9 (16.9%)		9 (37.5%)	
Biallelic	15 (28.3%)		15 (62.5%)	
Frameshift	1 (1.9%)		1 (4.2%)	
WHO Diagnosis				
None (n=18)				
OSP	10.2 ± 5.4	10.2 ± 5.4		
	9.75 (6.3 – 12)	9.75 (6.3 – 12)		
DCP	2.2 ± 0.9	2.2 ± 0.9		
	2 (2 – 2)	2 (2 – 2)		
MDS (n=14)				
OSP	42.0 ± 21.4	14.5 ± 7.8	46.6 ± 19.4	p=0.0444
	45.8 (25 – 55.0)	14.5 (9 – 20)	47.5 (28.3 – 58.3)	
DCP	32.7 ± 22.3	2 ± 0	37.8 ± 19.6	p=0.0283
	31.3 (15.3 – 41.7)	2 (2 – 2)	36.7 (22.3 – 47.9)	
AML (n=12)				
OSP	59.4 ± 29.9	53.3 ± 32.5	61.4 ± 30.8	p=0.6427
	60 (39.2 – 85)	55 (20 – 85)	65 (46.7 – 85)	
DCP	37.0 ± 27.5	12.5 ± 15.5	45.2 ± 26.1	p=0.0640
	32.5 (16.3 – 52.5)	5.2 (2 – 30.3)	44.2 (31.7 – 56.7)	
Other (n=9)*				
OSP	13.9 ± 14.2	11.0 ± 12.1	19.6 ± 19.2	p=0.6041
	6 (3.8 – 16.7)	6 (3.8 – 12)	16.7 (2 – 40)	
DCP	3.8 ± 5.0	2.2 ± 0.4	7.0 ± 8.7	p=0.4795
	2 (2 – 2)	2 (2 – 2)	2 (2 – 17)	

*CMML-1(2), prior acute monocytic leukemia with new *TP53* mutation (VAF 2.35%), and clonal hematopoiesis in plasma cell neoplasm, plasma cell neoplasm, MPN, systemic mastocytosis, MGUS, and T-cell clonal populations.”.

Three *TP53*-mutated cases with <5% DCP were unanimously identified by the six HPs. One case, categorized as AML with myelodysplastic changes and *TP53* mutation/inactivation, exhibited uniform cytoplasmic staining with <5% nuclear staining, but a high OSP. This case involved a frameshift *TP53* mutation (VAF 66.45%) and a complex karyotype, including a chromosome 17 deletion. Another case, diagnosed as AML in remission, showed no p53 staining, likely due to a very low VAF (2.35%) from a hotspot mutation (R158H) and a normal karyotype. The third case, classified as CMML-1 with a myelodysplastic subtype, had a missense mutation and a VAF of 22.2%.

**Figure 4:**
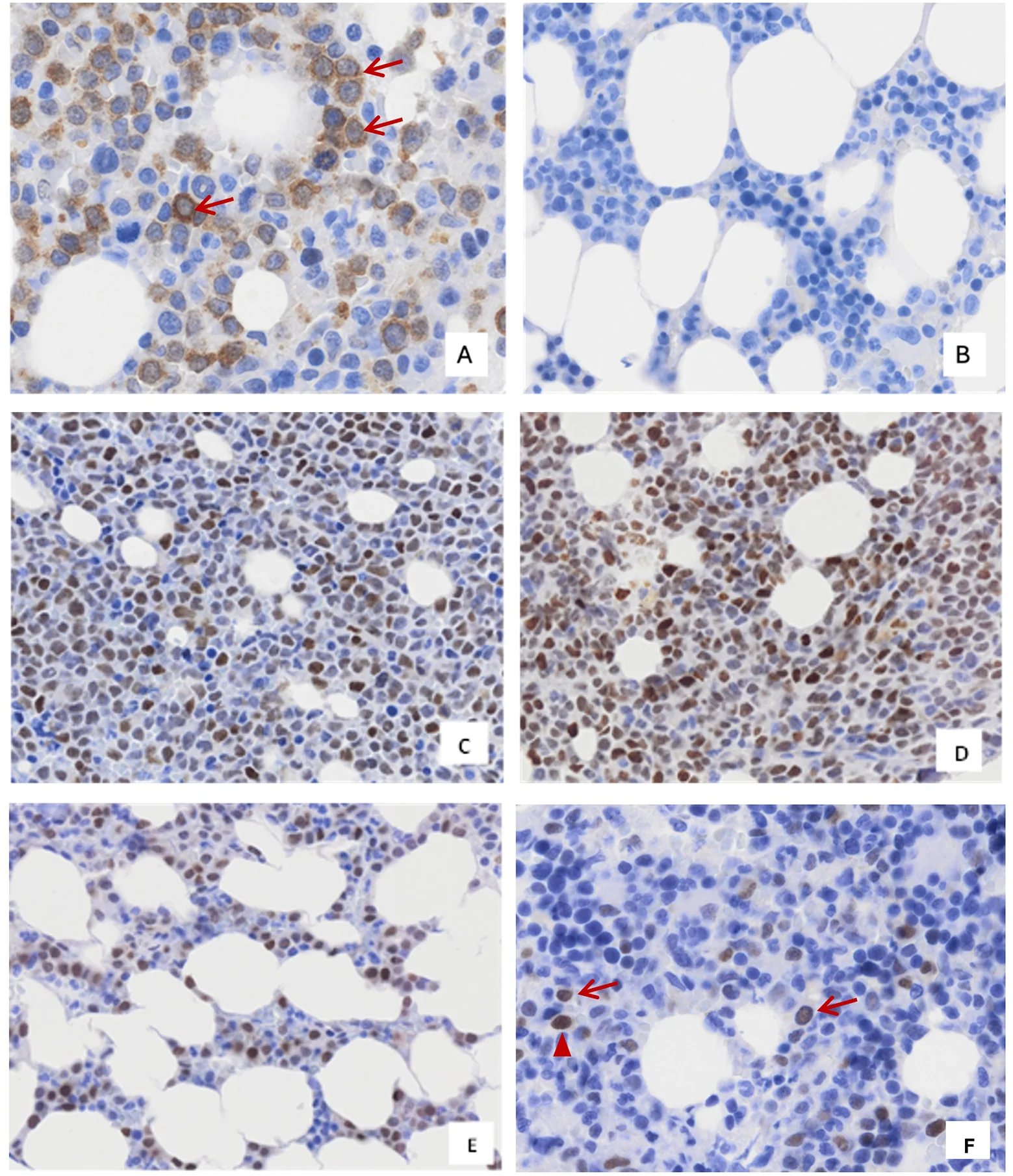
Spectrum of TP53 immunohistochemistry patterns in myeloid and plasma cell neoplasms. TP53-mutated AML-MDS shows cytoplasmic p53 with absent nuclear staining **(A)**, while TP53-mutated AML-MR demonstrates weak nuclear staining **(B)**. TP53–wild-type AML shows increased staining that may mimic TP53 mutation **(C)**. AML with KMT2A rearrangement and TP53 mutation displays variable nuclear staining intensity **(D)**. Plasma cell myeloma with low-VAF TP53 mutation shows heterogeneous staining with notable interobserver variability **(E)**. All images at 20× digital imaging.

Another case (**Figure 4**) with variable DCP ratings (<5% to 30%) involved a plasma cell neoplasm with a *TP53* mutation (VAF 2.37%) and clonal hematopoiesis of indeterminate potential (CHIP). This case exhibited a complex karyotype with a chromosome 17 deletion, 10% plasma cells in aspirate smears, and 40% by CD138 staining. Similarly, an AML case (**Figure 4**) with KMT2A rearrangement and a *TP53* mutation (VAF 13.87%) showed substantial variability in DCP estimates (5% to 70%), underscoring challenges in consistent interpretation among pathologists.

In the control group, most specimens had an average DCP of <10%, except for one case (**Figure 4**) with significant variability in DCP ratings (<5% to 50%). This case, diagnosed as AML without maturation, showed a very high OSP (80–90%), likely contributing to inter-observer variability.

These findings highlight the complexity of interpreting p53 IHC in cases with low DCP, high OSP, or low VAF, emphasizing the need for standardized interpretation and supplemental testing in challenging scenarios.

### DCP but not OSP is associated with *TP53*ms VAF

**Table 3** illustrates the relationship between VAF and the collective assessments of all six HPs for both stained cells and strongly stained cells. Our analysis showed that OSP estimates were not associated with VAF while DCP was.

**Table 3:** Pearson correlation coefficients between VAF and OSP and DCP.

Evaluator	Overall staining cells		Dark staining cells	
	Pearson correlation	P-value	Pearson correlation	P-value
Pathologist # 1	0.16017	0.4764	0.46425	0.0295
Pathologist # 2	0.21101	0.3459	0.33742	0.1246
Pathologist # 3	0.24433	0.2732	0.37890	0.0820
Pathologist # 4	0.39564	0.0684	0.53839	0.0097
Pathologist # 5	0.21679	0.3325	0.42980	0.0459
Pathologist # 6	0.25931	0.2439	0.53444	0.0104
Average of all Pathologists	0.25233	0.2573	0.46919	0.0276
Median of all Pathologists	0.28406	0.2001	0.51646	0.0139

## Discussion

*TP53* mutational status plays a crucial role in the prognosis and classification of myeloid neoplasms (2–4, 8). Accurate assessment of *TP53* genetic alterations is essential for guiding treatment decisions and predicting patient outcomes (10). While p53 IHC is a cost-effective and rapid surrogate for *TP53* genetic analysis, interpreting p53 IHC can be challenging due to wild-type expression patterns in normal cells (2–4, 8, 11, 12). The study demonstrated high inter-rater reliability among pathologists in evaluating p53 IHC using the monoclonal antibody DO-7 staining characteristics. Analysis of the DCP revealed substantial inter-observer agreement, with an overall ICC of 0.848. Stratification by *TP53*ms showed that the agreement remained substantial but was lower for WT-*TP53* cases (ICC = 0.644) than for *TP53*m cases (ICC = 0.806).

Different cutoffs for interpretation of p53 in cases of AML and MDS, have been described in the literature, with suggested cutoffs between <5 and <10% (2, 4, 8, 13). Accordingly, the cases studied were evaluated using both OSP and DCP and dichotomized into <5% and >5%, as well as <10% and >10%. Comparing the data with <5% with >5% for OSP and DCP, the six HPs achieved moderate and substantial agreement, respectively (Kappa=0.433 and 0.872, respectively). By increasing the cutoff to <10% and >10% the findings were similar (Kappa=0.552 for OSP and 0.893 for DCP). The agreement between raters was strong for samples with and without *TP53* mutations, with Kappa values of 0.902 and 0.833, respectively. This robust agreement in DCP ensures reproducibility in the interpretation of p53 IHC based on this cohort. When looking at MDS diagnosis, the 5% cutoff is effective regardless of *TP53*ms with a marginally higher agreement in the mutated samples. This might be due to the more distinctive staining patterns often observed in *TP53*m cases, which could be easier to consistently assess. The Kappa results of <10% and >10%, showed better results for AML cases as demonstrated in **Table 4**. These findings are in agreement with previous studies (3, 4, 8, 13) suggesting p53 staining for MDS vs. AML cutoff values of 5% and 10%, respectively.

**Table 4:** Kappas within WHO diagnosis groups (<10% vs >10%).

WHO Diagnosis	*OSP*	*DCP*
AML (n=12)	1.0 (p<0.001)	0.852 (p<0.001)
MDS (n=14)	0.101 (p=0.391)	0.722 (p<0.001)
Other (n=9)	0.500 (p<0.001)	0.780 (p<0.001)

Applying a 10% cutoff for DCP, regardless of the diagnosis, yielded better results, with a sensitivity of 87.5%, specificity of 96.6%, PPV of 95.4%, and NPV of 90.3%. These higher values for DCP suggest it is a more effective predictor of *TP53* mutations than OSP as demonstrated in other studies (2, 4, 8, 11). The stain used in this study was validated in our laboratory for both decalcified bone marrow core biopsies and non-decalcified clot sections. No apparent differences in staining quality or interpretation were observed between specimen types; however, formal comparative analyses were not performed. Future studies are needed to evaluate potential differences in diagnostic performance between these specimen types.

The ROC curves for OSP and DCP demonstrated strong area AUC values, also supporting the high diagnostic accuracy of p53 IHC in predicting *TP53* mutations. When applying a 30% cutoff for OSP, the sensitivity was 83.3%, specificity was 89.7% (**Figure 3** PPV was 87.0%, and NPV was 86.7%.

In MDS and AML, *TP53* mutations are associated with adverse prognosis and have important diagnostic and prognostic implications. Therefore, a rapid p53 immunohistochemical result may provide useful preliminary information while awaiting confirmatory molecular testing. Although p53 immunohistochemistry may facilitate earlier recognition of cases likely to harbor *TP53* alterations, our study did not evaluate treatment response or clinical outcomes, and molecular testing remains essential for definitive diagnosis, risk stratification, and therapeutic decision-making. Given the high specificity (>96%) of DCP >10%, a strongly positive IHC result may increase confidence in the presence of a *TP53* while awaiting molecular confirmation.

These findings support the use of p53 IHC as a rapid, cost-effective surrogate marker for TP53 mutations in MDS, in resource constrained settings especially when molecular testing is unavailable or significantly delayed. In contrast to MDS, neither OSP nor DCP significantly distinguished *TP53*-mutated from *TP53* wild-type AML cases in our cohort. Previous studies have shown that p53 immunohistochemical expression in AML is affected by *TP53* mutation subtype, variant allele frequency, and allelic status, with lower sensitivity for truncating mutations and variable expression among biologically distinct AML subgroups. These factors, together with coexisting cytogenetic abnormalities and the limited number of AML cases in our cohort, likely contributed to our findings (4, 8, 11).Therefore, p53 immunohistochemistry should be considered an adjunct to molecular testing in AML. These findings emphasize the need for complementary molecular techniques, such as NGS in AML.

When evaluating other myeloid neoplasms, the results were inconsistent and likely due to low number of cases with diagnoses other than MDS and AML. Additional studies are needed to evaluate the prognostic significance of p53 staining in specific myeloid neoplasms such as MPN and MDS/MPN. This supports a targeted approach to using IHC in cases where *TP53* status has actionable implications, rather than applying it broadly across all myeloid malignancies. Future studies in larger, disease-specific cohorts are needed to validate and refine these cutoff values, determine whether entity-specific thresholds are more appropriate, and improve the generalizability of the findings.

One example of a challenge in staining interpretation was a case in the *TP53* mutated group (**Figure 4**), which showed a unique diffuse cytoplasmic staining pattern. This was a case of AML, myelodysplasia-related (WHO and ICC), with a frameshift *TP53* mutation. This pattern of staining has been reported in other neoplasms in pelvic and endometrial carcinomas (14–17). However, to the best of our knowledge, this pattern of staining and its significance has not yet been established in hematopoietic neoplasms.

In the evaluation of the staining intensity and the VAF correlation, the findings suggest that while the OSP provides some insight, the DCP showed a moderate correlation of 0.469 between VAF and DCP staining (see **Table 3**), which is most likely due to the complexity of *TP53* mutation/inactivation and the interpretation of p53 staining and our diverse set of diagnosis. Consistent with previous studies, DCP demonstrated a moderate positive correlation with *TP53* variant allele frequency (r = 0.469), suggesting that p53 protein expression generally increases with increasing mutant allele burden, although additional biological factors also contribute to staining variability (4, 18). In addition to the correlation with VAF, it is important to consider *TP53* deletions by karyotype, FISH or loss-of-heterozygosity studies. This was demonstrated in a case of plasma cell neoplasm with a low VAF (2.37%) and a complex karyotype with loss of chromosome 17. It is possible that the staining of p53 was predominantly in plasma cell areas which caused difficulties in the interpretation of p53 staining within plasma cells or other hematopoietic cells.

Missense mutations were prevalent among the cohort of *TP53* mutation cases, with notable hotspot mutations including p.(R175H), p.(R273H), p.(R273C), and p.(G245S) (19).Each of these cases demonstrated dark staining of >5%. The existence of these known hotspot mutations has brought forward possible targets for future therapies (19, 20). As expected, most of the *TP53*m cases had complex karyotypes, and 9/24 cases exhibited deletions of chromosome 17 by karyotype, confirming the already known genetic and cytogenetic characteristics associated with *TP53* mutations and genomic instability (3, 9, 21). Another limitation of this study is that copy-neutral loss of heterozygosity (cnLOH) was not evaluated. Because cnLOH is a recognized mechanism of biallelic TP53 inactivation, reliance on variant allele frequency, chromosome 17 deletion, and conventional cytogenetics may have underestimated the frequency of multi-hit TP53 alterations in our cohort (21). Future studies incorporating methods capable of detecting cnLOH may provide a more comprehensive assessment of *TP53* allelic status. These cases encompassed a spectrum of diagnoses, including AML with myelodysplasia-related changes (29% of *TP53* mutated cases), MDS with biallelic *TP53* inactivation (29%), and other hematologic neoplasms, such as plasma cell neoplasms. The prevalence of specific hematologic diagnoses within the *TP53*-mutated group further evidences the association between *TP53* mutations and disease pathogenesis. Specifically, myelodysplasia-related conditions and post-cytotoxic diagnostic qualifiers were prominently represented in our cohort.

This study has several limitations. The cohort included a heterogeneous spectrum of myeloid neoplasms, clonal hematopoiesis, plasma cell neoplasms, and non-neoplastic bone marrow specimens, reflecting our institution’s routine clinical practice. While this broad case selection enhances the “real-world” applicability of our findings, it may also have influenced the reported diagnostic performance, ROC analysis, and optimal cutoff values due to prevalence of *TP53* mutation and TP53 expression within the neoplasms studied. Consequently, the proposed cutoff values should be interpreted within the context of this mixed cohort and may not be directly generalizable to individual disease categories. Larger disease-specific validation studies, particularly in homogeneous cohorts such as MDS or AML, are warranted to refine these thresholds.

In summary, this study uniquely emphasizes the high inter-observer reliability of p53 IHC interpretation between six HPs for the assessment of *TP53* mutations in a diverse set of myeloid cases. Different cutoffs for interpretation of p53 staining with values of 5% and 10% should be applied in diagnosis of MDS or AML, respectively. Although pathologists may encounter challenges in their interpretation, p53 serves as a quick and cost-effective surrogate diagnostic tool until the NGS results are available. Future research should explore different DCP thresholds across hematologic malignancies and consider automated or AI-assisted image analysis to improve the diagnostic accuracy and possibly aid therapeutic advances in clinical practice.

## Data Availability

The original contributions presented in the study are included in the article/supplementary material. Further inquiries can be directed to the corresponding authors.
